# Airway epithelial cells mount an early response to mycobacterial infection

**DOI:** 10.3389/fcimb.2023.1253037

**Published:** 2023-09-26

**Authors:** Amy M. Barclay, Dennis K. Ninaber, Suzanne van Veen, Pieter S. Hiemstra, Tom H. M. Ottenhoff, Anne M. van der Does, Simone A. Joosten

**Affiliations:** ^1^ Department of Infectious Diseases, Leiden University Medical Center, Leiden, Netherlands; ^2^ Department of Pulmonology, Leiden University Medical Center, Leiden, Netherlands

**Keywords:** tuberculosis, airway epithelium, host-pathogen interaction, air-liquid interface (ALI) 3D *in vitro* model, non-tuberculous mycobacteria

## Abstract

Lung epithelial cells represent the first line of host defence against foreign inhaled components, including respiratory pathogens. Their responses to these exposures may direct subsequent immune activation to these pathogens. The epithelial response to mycobacterial infections is not well characterized and may provide clues to why some mycobacterial infections are cleared, while others are persistent and pathogenic. We have utilized an air-liquid interface model of human primary bronchial epithelial cells (ALI-PBEC) to investigate the epithelial response to infection with a variety of mycobacteria: *Mycobacterium tuberculosis* (Mtb), *M. bovis* (BCG), *M. avium, and M. smegmatis*. Airway epithelial cells were found to be infected by all four species, albeit at low frequencies. The proportion of infected epithelial cells was lowest for Mtb and highest for *M. avium*. Differential gene expression analysis revealed a common epithelial host response to mycobacteria, including upregulation of *BIRC3, S100A8* and *DEFB4*, and downregulation of *BPIFB1* at 48 h post infection. Apical secretions contained predominantly pro-inflammatory cytokines, while basal secretions contained tissue growth factors and chemokines. Finally, we show that neutrophils were attracted to both apical and basal secretions of infected ALI-PBEC. Neutrophils were attracted in high numbers to apical secretions from PBEC infected with all mycobacteria, with the exception of secretions from *M. avium*-infected ALI-PBEC. Taken together, our results show that airway epithelial cells are differentially infected by mycobacteria, and react rapidly by upregulation of antimicrobials, and increased secretion of inflammatory cytokines and chemokines which directly attract neutrophils. Thus, the airway epithelium may be an important immunological component in controlling and regulating mycobacterial infections.

## Introduction

Tuberculosis (TB) is caused by the highly successful respiratory pathogen *Mycobacterium tuberculosis* (Mtb), which has infected approximately a quarter of the world population (Paulson, 2013). For decades, Mtb has been the leading cause of death from a single infectious agent, killing over 1.5 million people each year (Global tuberculosis report, 2022). Despite enormous efforts, there is currently no effective vaccine against TB apart from the Bacille Calmette-Guérin (BCG) vaccine, which offers only partial protection (Fine, 1995; Foster et al., 2021). In addition, the emergence of multi-drug resistant Mtb strains further heightens the impact of TB worldwide.

TB is generally considered a respiratory disease, as the lung is the primary site of infection in most patients. Via coughing, patients with active TB produce small aerosols containing the pathogen, which are subsequently inhaled by another host. Once inhaled, these aerosols pass through the upper airways into the lower airways and alveoli. Healthy human airways are lined with epithelial cells which are in direct contact with the air we breathe as well as the numerous foreign particles it contains. Due to this abundance of epithelial cells, it is likely that inhaled mycobacteria first encounter airway epithelial cells.

Several studies showed that Mtb interacts with epithelial cells in the airways and alveoli (reviewed by de Waal et al. (2022)). For example, epithelial cells of the bronchi and alveoli were able to sense mycobacteria via Toll- and NOD-like receptors, and in response secrete a variety of pro-inflammatory cytokines and antimicrobial peptides (Leiva-Juarez et al., 2018; Johnston et al., 2021). Epithelium-derived cytokines attract immune cells such as neutrophils (Nouailles et al., 2014), but also signal to antigen-presenting dendric cells (Yang et al., 1999). Furthermore, bronchial and alveolar epithelial cell lines could be infected by different species of mycobacteria, including Mtb (Bermudez and Goodman, 1996; Birkness et al., 1999; Reuschl et al., 2017; Matsuyama et al., 2018). This indicates the lung epithelium might play a role in mycobacterial infections, for example as sensors of infection or as a niche allowing mycobacterial growth. However, epithelial responses have been only partly characterized, and only few studies have included comparisons between mycobacterial species. Furthermore, most studies utilized cell lines or submerged undifferentiated epithelial cultures, which resemble the human airways only to a limited extent, as they do not contain all relevant epithelial cell types such as basal, ciliated, goblet and club cells. To accurately model the airway epithelium, air-liquid interface (ALI) models are especially relevant. In such models, primary bronchial epithelial cells (PBEC) are exposed to air on one side, which not only mimics the physiological situation, but also induces differentiation into a pseudostratified epithelium similar to those in human lungs (Hiemstra et al., 2018; Upadhyay and Palmberg, 2018; Hiemstra et al., 2019). Another aspect that has not been studied are epithelial responses to different mycobacterial strains with variable virulence.

Here, we characterized responses of well-differentiated PBEC to various mycobacterial strains. We infected these ALI-PBEC cultures with 4 different mycobacterial strains, including pulmonary pathogens *M. tuberculosis* (Mtb) and *M. avium*, the vaccine strain *M. bovis* (BCG) and the non-pathogenic *M. smegmatis*. These strains represent a range in virulence, with Mtb being the most virulent, followed by *M. avium*, BCG, and finally the commensal *M. smegmatis.* We determined epithelial infection rates for each of these species, characterized the epithelial host response by measuring cytokine production and gene expression, and assessed the functional neutrophil recruitment by cytokine secretions from infected PBEC.

## Methods

### Bacteria

Mycobacteria were grown in Difco Middlebrook 7H9 medium (BD Biosciences, Franklin Lakes, NJ, USA) containing 0.05% Tween-80 (Sigma-Aldrich, St. Louis, MO, USA), 0.5% glycerol (Sigma-Aldrich), and 10% Middlebrook ADC growth supplement (750 mM BSA + 110 mM dextrose + 130 µM catalase in H_2_O, produced in our lab). A list of bacterial strains used, and their origins can be found in **Table S1**. The *M. avium* strain expressing Wasabi, the *M. bovis* BCG strain expressing GFP, and the *M. smegmatis* strain expressing GFP were cultured with 100 µg/ml hygromycin B (Invitrogen, Thermo Fisher Scientific, Waltham, MA, USA). The Mtb H37Rv strain expressed Venus constitutively. All strains were diluted to OD_600 = _0.25 the day before experiments were performed, to ensure exponential growth of the bacteria.

Batches of heat-killed mycobacteria were prepared; OD_600_ was measured, and bacteria were heated to 56°C for 30 minutes. Heat-killed bacteria were then diluted to a multiplicity of infection (MOI) of 100 in epithelial cell culture medium (see below) without antibiotics. Aliquots of heat-killed bacteria were stored at -20°C.

### Bronchial epithelial cell culture

Primary bronchial epithelial cells (PBEC) were isolated from macroscopically normal lung tissue obtained from patients undergoing resection surgery for lung cancer at the Leiden University Medical Center, the Netherlands. Patients from which this lung tissue was derived were enrolled in the biobank via a no-objection system for coded anonymous further use of such tissue (www.coreon.org). However, since 01-09-2022, patients are enrolled in the biobank using active informed consent in accordance with local regulations from the LUMC biobank with approval by the institutional medical ethical committee (B20.042/Ab/ab and B20.042/Kb/kb). Cells were cultured as previously described (Schrumpf et al., 2020). In brief, PBEC (passage 1) of individual donors were expanded for 5 days in T75 culture flasks pre-coated with a mixture of 30 μg/ml Purecol (Advanced BioMatrix, Carlsbad, CA, USA), 5 μg/ml human fibronectin (PromoCell, Heidelberg, Germany) and 10 μg/ml BSA (Fraction V; Thermo Fisher Scientific, Carlsbad, CA, USA). Per experiment, mixes of 4 unique donors were prepared (Thaler et al., 2023): PBEC of 2 male and 2 female donors, all non-COPD patients, were mixed, and seeded at equal concentrations and at a high density of 160.000 cells on pre-coated 0.4 µm membrane pore size Transwell inserts (Corning Costar, Corning, NY, USA) in 12-well plates to rapidly obtain confluence and prevent excessive growth of a certain donor in the mix. Cells were cultured in medium consisting of one part Bronchial Epithelial Cell Medium-basal (BEpiCM-b (ScienCell, Carlsbad, CA, USA)) and one part Dulbecco’s modified Eagle’s medium (DMEM (Gibco, Thermo Fisher Scientific)) supplemented with Bronchial Epithelial Cell Growth Supplement (BEpiCGS; ScienCell), 12.5 mM HEPES (Gibco, Thermo Fisher Scientific), 100 U/ml penicillin, 100 ug/ml streptomycin (all from ScienCell), 2 mM glutaMAX (Thermo Fisher Scientific), further referred to as complete BD-medium (cBD). In the submerged stage, cBD was supplemented with 1 nM EC-23 (Tocris, Bristol, UK). After reaching 100% confluence, apical medium was removed, and cells were differentiated at the air-liquid interface (ALI) in cBD supplemented with 50 nM EC 23. Culture medium was changed 3 times per week during which the apical side of the culture was carefully washed with 200 µl warm PBS for 10 min at 37°C to wash away mucus and cell debris. After 14 days of mucociliary differentiation at ALI, all dominant cell-types were present and ciliary activity was observed. Cultures were subsequently used for experiments.

### Infection of airway epithelial cell cultures

ALI-PBEC were cultured using cBD medium without antibiotics 2-4 days before infection experiments. Mtb Venus, BCG GFP, *M. avium* Wasabi and *M. smegmatis* GFP were grown as described above. Although the addition of 0.05% Tween-80 to bacterial cultures minimized bacterial aggregation, clumping cannot be completely ruled out. OD_600_ of visually homogenous bacterial cultures was measured. Bacterial suspensions at predicted multiplicities of infection (MOI) of 10 and 100 were prepared accordingly in cBD medium supplemented with 50 nM EC-23. For these calculations, PBEC cell density was estimated at 1x10^6^ cells per insert. Bacterial suspensions were plated on 7H10 agar to retrospectively determine the actual infection load. Mucus was washed from the apical surface of ALI-PBEC cultures with warm PBS for 10 min at 37°C. Immediately following mucus removal, 50 µl bacterial suspension was carefully added to the apical side of the cells. Control cultures received medium without bacteria. For gene expression and Luminex assays, heat-killed bacteria were used in parallel with live bacteria. Next, cultures were centrifuged briefly at 300 x g for 2 min to spin the bacteria down onto the cell layer, and incubated for 24 h at 37°C. Then, 150 µl PBS was added to the apical side and cultures were incubated 15 min at 37°C. From each ALI-PBEC insert, 200 µl apical supernatant and 1 ml basal medium was collected and stored at -20°C for use in Luminex and neutrophil migration assays. The remaining cBD was changed with 1 ml new medium, and apical sides were treated with 30 µg/ml gentamicin (Sigma-Aldrich) for 15 min at 37°C to kill extracellular bacteria. Next, apical sides of cultures were washed with PBS, and cultures were incubated overnight at 37°C. At this point, cultures were used for the various assays described below.

### Flow cytometry

Basal medium from ALI-PBEC cultures was replaced with calcium-free PBS and 200 µl was added to the apical side as well. Cultures were incubated 30 min at 37°C. PBS was removed, and 200 µl pre-warmed TrypLE Express (Gibco, Thermo Fisher Scientific) containing 10 µM Y-27632 (StemCell Technologies, Vancouver, BC, Canada) was added onto the cell layer. Cultures were incubated for 15 min at 37°C. After pipetting up and down to break up the cell layer, single cells were collected from the inserts and transferred to tubes containing 800 µl PBS. Of each sample, 100 µl of the total volume was transferred to a separate tube for the colony forming units assay (see below), the remaining cells were used for flow cytometry. Samples were stained with Violet Fixable Live/Dead dye (Invitrogen, Thermo Fisher Scientific), 1:800 in PBS for 30 min at RT, then washed with PBS. Samples infected with *M. avium*, BCG or *M. smegmatis* were fixed in 4% paraformaldehyde (PFA) (Alfa Aesar, Thermo Fisher Scientific) for 1 h, then resuspended in PBS with 0.1% w/v BSA. Flow cytometry was performed with a Cytek^®^Aurora (Cytek Biosciences, Fremont, CA, USA) cytometer at the Flow cytometry Core Facility (FCF) of Leiden University Medical Center (LUMC) in Leiden, Netherlands (https://www.lumc.nl/research/facilities/fcf). Due to bio-safety level restrictions, samples infected with *M. tuberculosis* were fixed in 4% PFA for 10 minutes, then measured in fixative with a BD FACS Lyric 3L12C (BD Biosciences) cytometer in the BSL-3 lab.

### CFU assay

Single cell samples collected in tubes during sample preparation for flow cytometry were treated with 30 µg/ml gentamicin (Sigma-Aldrich) for 15 min at 37°C to kill extracellular bacteria, then washed with PBS. Cells were lysed in H_2_O + 0.05% SDS (Invitrogen, Thermo Fisher Scientific) for 15 min at RT. Then those lysates were washed with PBS, centrifuged at 800 x g and resuspended in 100 µl PBS. Serial dilutions of the lysates were plated on Difco 7H10 agar (BD Biosciences) plates and incubated at 37°C. The number of colonies formed was determined by manual count.

### Confocal microscopy

Following infection, ALI-PBEC cultures were washed with PBS on both sides to remove mucus and cellular debris, and remove any remnants of culture medium. Cultures infected with *M. avium*, BCG or *M. smegmatis* were fixed in 4% PFA for 1 h at 4°C, while cultures infected with *M. tuberculosis* were fixed in 4% PFA for 24 h at 4°C due to safety regulations. Cultures were washed with and stored in PBS at 4°C until staining. Cell culture inserts were blocked with PBS containing 1% BSA and 0.3% Triton X-100 (Fluka Chemie, Buchs, Switzerland) for 10 min at RT. The PET membranes were removed from the plastic holders with a scalpel, and cut in two. The membrane halves were submerged in primary antibody solution (**Table S2**) for overnight incubation at 4°C. Membranes were washed 3 times with PBS before incubation with secondary antibodies (**Table S2**) and DAPI for 2 h at 4°C. Membranes were washed 3 times in PBS and 3 times in demi water, then placed on a glass slide, and mounted in ProLong Diamond Antifade mountant (Invitrogen, Thermo Fisher Scientific). All samples were imaged with an SP8 confocal microscope (Leica, Wetzlar, Germany) at the Light and Electron Microscopy Facility of the Leiden University Medical Center in Leiden, Netherlands (https://www.lumc.nl/research/facilities/light-and-electron-microscopy-facility/services/). Image processing was performed in Leica Application Suite X (LAS X) software.

### RNA isolation and microfluidic multiplex qPCR gene expression assay

ALI-PBEC cultures were stimulated with live or heat-killed bacteria. At the relevant time point, cultures were washed with PBS to remove mucus and cellular debris. The cell layer was lysed with 350 µl TRIzol (Ambion, Foster City, CA, USA) for 5 min at RT. All liquid was collected into microtubes and stored at -80°C awaiting processing. To isolate RNA, samples were thawed on ice, incubated with 70 µl chloroform (EMSURE, Merck, Rahway, NJ, USA) for 3 min, and centrifuged at 12.000 x g for 15 min at 4°C. The aqueous phase of each sample was transferred to microtubes with 175 µl isopropanol (Supelco, Bellefonte, PA, USA), incubated 10 min at RT, and centrifuged. Supernatant was removed and the pellet was resuspended in 350 µl 75% ethanol (EMSURE, Merck). Samples were centrifuged and all ethanol was removed. Isolated RNA was then dissolved in nuclease-free H_2_O (Ambion). RNA was stored at -80°C until use in the gene expression assay.

RNA was treated with DNA-free DNA Removal Kit (Invitrogen, Thermo Fisher Scientific) according to manufacturer’s instructions. RNA concentration was measured with a DS-11 spectrophotometer (DeNovix, Wilmington, DE, USA) and samples were diluted to 50 ng/µl. cDNA was synthesized using Reverse Transcription Master Mix (Fluidigm, South San Francisco, CA, USA), according to manufacturer’s instructions. The cDNA was stored at -20°C. A set of 94 genes was selected to broadly analyse the host response. These included genes involved in pathogen sensing, inflammation, epithelial-to-mesenchymal transition (EMT), and also genes encoding antimicrobials and cell type markers. A pool of these 94 primer pairs (**Table S3**) was created in DNA suspension buffer (10 mM Tris, pH 8.0, 0.1 mM EDTA). For pre-amplification reactions, PreAmp Master Mix (Fluidigm) was combined with the primer pool and the cDNA previously obtained, and thermal cycled according to manufacturer’s instructions. Reactions were cleaned with Exonuclease I (Thermo Fisher Scientific) treatment. Cleaned pre-amplified cDNA was diluted 5 times in DNA suspension buffer. Target assay and sample pre-mixes were prepared to load into the Integrated Fluidic Circuit (IFC) chip (Fluidigm). Assay pre-mixes each contained 0.4 µl of respective primer pairs, 1.6 µl DNA suspension buffer and 2 µl Assay Loading Reagent (Fluidigm). Sample pre-mixes each contained 2 µl SsoFast Evagreen Supermix (BioRad, Hercules, CA, USA), 0.2 µl Sample Loading Reagent (Fluidigm) and 1.8 µl pre-amplified ExoI-cleaned cDNA. A 96x96 IFC chip (Fluidigm) was primed and assay and sample pre-mixes were loaded. The chip was pressurized using the HX IFC controller and run on the Biomark HD (qPCR) system according to manufacturer’s instructions. During analysis, data points with a cycle threshold (Ct) value over 35 were considered at the cutoff for reliable detection and were set to Ct 35. Relative target gene expression (ΔCt) was calculated by subtracting the geometric mean of three most stable reference genes (determined using geNorm from the *ctrlGene* r package): *RPL13A, ATP5B and B2M*.

### Luminex assay

After 24h infection, 200 µl apical supernatant and 1 ml basal medium were collected from each cell culture and stored at -20°C until processing. These were filtered in FiltrEX 96-wells filter plates (Corning Costar) with pore size 0.2 µm to remove bacteria. Supernatants and medium were diluted 4 times with Luminex Assay buffer (Bio-Rad, Hercules, CA, USA). Following manufacturer’s instructions, the Bio-Plex Pro Human Cytokine 27-plex Assay (Bio-Rad) was performed. Samples were measured on a Bio-Plex 200 System (Bio-Rad). Per analyte, a lower and upper limit of detection was determined with standard curves. Concentrations measured below the assays’ detection limit were set to 1 pg/ml, and those measured over the detection limit were set to the maximum quantifiable pg/ml per analyte. As apical wash was collected in a 5 times smaller volume than basal medium, concentrations retrieved in the groups were corrected for differences in sample volume before further analysis.

### Neutrophil migration assay

Apical and basal supernatants from infected ALI-PBEC (see Infection of airway epithelial cell cultures above) were pooled to obtain sufficient volume to assess functional neutrophil recruitment: supernatants from live- and heat-killed-stimulated ALI-PBEC were combined together, and then respective samples from all 5 donor pools were combined as well. A schematic of how samples were pooled can be found in **Supplementary Figure 4A**. Pooled apical supernatants were then diluted 1:1 in PBS to obtain a sufficient volume for this assay.

Whole blood from 3 healthy donors enrolled in the LUMC Voluntary Donor Service (LuVDS) was collected in two 10 ml heparin blood collection tubes. All donors gave broad informed consent. The biomaterial of these donors was released for research purposes only, and its use was approved by the Institutional Review Board (LuVDS23.007). Each tube of whole blood was transferred to a 50 ml tube and diluted with 15 ml PBS. Next, 10 ml Ficoll (LUMC Hospital Pharmacy, Leiden, Netherlands) was added at the bottom of each tube, which were then centrifuged for 20 min at 650 x g. Supernatant was discarded until only a pellet containing neutrophils and erythrocytes was left. To lyse erythrocytes, pellets were resuspended in 40 ml lysis buffer (0.1 mM EDTA, 180 mM NH_4_Cl, 10 mM KHCO_3 in_ H_2_O, pH 7.4). The moment the suspension turned dark red after approx. 2-5 minutes, tubes were immediately centrifuged for 10 min at 290 x g. Supernatant was discarded, pellets were combined into 1 tube and resuspended in 45 ml PBS, then centrifuged 10 min at 290 x g. This washing step was repeated once. Cell pellets were resuspended in 1-2 ml RPMI supplemented with 20 mM HEPES (Gibco), and cells were counted manually. Cell suspension was diluted to 7.5x10^5^ cells/ml in RPMI with HEPES. Then, 3.0 µm inserts (Greiner Bio-one, Alphen aan den Rijn, Netherlands) were placed in a 12-well culture plate and treated with 400 µl blocking solution (PBS containing 0.1% w/v BSA) for 15 min at RT. Blocking solution was removed from the inserts, and 1 ml pooled supernatant from infected ALI-PBEC was added to each basal compartment. As a positive control, 10 nM fMLP (Sigma-Aldrich) and 10% fetal bovine serum (Corning) in cBD medium (to compare to basal supernatants) or in PBS (to compare to apical supernatants) was used. Of the neutrophil cell suspension, 400 µl was added on top of each blocked insert. Neutrophils were allowed to migrate through inserts for 1.5 hours at 37°C. After incubation, 50 µl of 50 mM EDTA was added to basal compartments. After 2 min, inserts were discarded. From basal compartments 500 µl liquid was transferred to FACS tubes. From each sample, the number of neutrophils in 100 µl was measured on an Accuri C6 Plus (BD Biosciences) flow cytometer.

### Statistics

Data analysis was performed in Graphpad Prism version 9.3.1, and in R Studio version 4.1.2. R packages used were: caret, tidyverse, tidyr, ggplot2, reshape2, viridis, plotrix, ggrepel, mixOmics, scales, dplyr, and ctrlGene. All data were considered non-parametric. Differences in infection percentages were tested with the Friedman test + Dunn correction for multiple testing. Differentially expressed genes (DEGs) were determined by calculating log2 fold change by -ΔΔCt-method, subtracting the median relative expression (ΔCt) of the reference group from that of the target group and multiplying by -1. Threshold for statistical significance of DEGs was p < 0.05. Statistics were performed using the Mann-Whitney U test. Due to the small sample size and the resulting low statistical power, no corrections for multiple testing were performed on gene expression data. Differentially secreted cytokines were determined with the Mann-Whitney U test with FDR correction (Benjamini-Hochberg) for multiple testing. Clustering of gene expression and cytokine secretion profiles was done with the one minus Spearman rank correlation method.

## Results

### Bronchial epithelial cells are directly infected by mycobacteria

**Figure 1:**
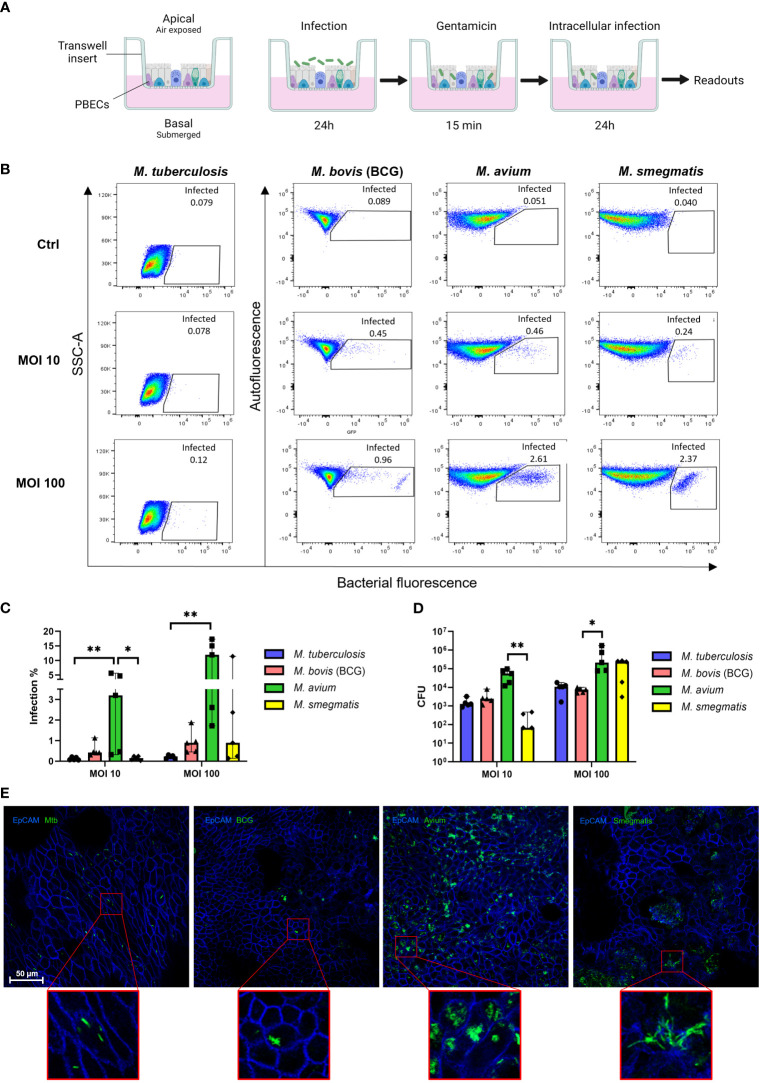
Well-differentiated bronchial epithelial cells are directly infected by various mycobacterial species. **(A)** Overview of air-liquid interface model and experimental setup. Created with BioRender.com **(B)** Representative result of one flow cytometry experiment. Gated population represents infected cells. **(C)** Infection rates per bacterial species in air-liquid interface-cultured primary bronchial epithelial cells (ALI-PBEC), measured by flow cytometry. Shown as median + 95% confidence interval (CI). Statistics performed as Friedman test with Dunn’s correction for multiple testing. **(D)** CFU counts per ALI-PBEC donor pool, representing intracellular bacterial burden. Shown as median + 95% CI. Statistics performed as Friedman test with Dunn’s correction for multiple testing. **(E)** Confocal microscopy images of infected ALI PBEC models. EpCAM membrane marker depicted in blue, bacteria depicted in green. P<0.05 is indicated by *. P<0.01 is indicated by **.

To assess whether Mtb, *M. avium*, BCG, and *M. smegmatis* could infect human bronchial airway epithelial cell cultures and compare potential differences in infection percentages between these strains, well-differentiated PBEC were infected with the four different fluorescent mycobacterial strains for 24 h, followed by a short gentamicin treatment of 15 min to kill remaining extracellular bacteria. ALI-PBEC were then incubated for an additional 24 h before further analyses (**Figure 1A**). Following these 48 h, cultures were harvested, and the percentage of infected cells was measured by flow cytometry. The gating strategy is depicted in **Figure S1A**. The various mycobacterial strains infected epithelial cell cultures to a different extent (**Figure 1B**). The percentage infected cells depended on both the multiplicity of infection (MOI) and the bacterial strain. Due to BSL-3 restrictions, samples infected with Mtb were analysed on a different type of flow cytometer. To confirm that differences between pathogens were not the result of alternative flow cytometers, we also analysed samples from BCG, *M. avium* and *M. smegmatis*-infected ALI-PBEC models in the flow cytometer in the BSL-3 laboratory and obtained identical results (**Figures S1B–D**).

Combining data from all independent donor mixes, the highest infection percentage was observed for *M. avium*, followed by BCG and *M. smegmatis*, whereas the percentage of epithelial cells infected with Mtb was lowest (**Figure 1C**). Interestingly, the efficiency of *M. smegmatis and M. avium* infection in epithelial cells seemed to be at least partly dependent on the donor pool (**Figures S1E, F**). In the case of *M. smegmatis* infections, this donor mix-dependent variation in infection percentage was only observed at a high MOI of 100, whereas with *M. avium* this was seen at high and low MOIs.

The total intracellular mycobacterial burden per ALI-PBEC model, independent of bacterial fluorescence, was determined by colony forming unit (CFU) assays (**Figure 1D**). Similar to the flow cytometry read out, intracellular *M. avium* content was highest when comparing all tested mycobacterial strains. Intracellular *M. smegmatis* CFUs were highly dependent on MOI, while intracellular Mtb and BCG content was relatively low overall.

To confirm that mycobacteria were present intracellularly, infected ALI-PBEC cultures were stained with an antibody against the epithelial cell membrane marker EpCAM. Fluorescent bacteria of all four species were observed intracellularly by confocal microscopy (**Figure 1E**). *M. avium-*infected cultures again displayed the highest bacterial burden, with most infected cells per area, and often multiple bacteria per individual cell. In contrast, Mtb and BCG-infected cultures contained far fewer infected cells per area. Cultures infected with *M. smegmatis* generally showed more infected cells than Mtb- and BCG-infected cultures, but fewer than *M. avium-*infected ones. Infected cells were detected mostly on the apical side of ALI-PBEC cultures, and bacteria had not yet penetrated deeper layers. Finally, infection of ALI-PBEC cultures was rather heterogeneous, with not all areas of the epithelial culture having the same number of infected cells. Images shown in **Figure 1E** are of areas with high numbers of infected cells, from the top plane of each model which was defined as the first apical 1-5 µm of the epithelial culture.

Taken together, we demonstrate that well-differentiated epithelial cell cultures can be infected intracellularly by four mycobacterial species, albeit with varying efficiency.

### Mycobacterial infection induced a common gene expression pattern in PBEC

To explore the transcriptional response of airway epithelial cells induced by mycobacteria, ALI-PBEC were infected with Mtb, BCG, *M. avium* or *M. smegmatis* at MOI 100 (or the heat-killed equivalent thereof), after which changes in gene expression were assessed. Differential gene expression was assessed using microfluidic multiplex qPCR, including 94 genes involved in pathogen sensing, cytokine and antimicrobial mediator production, cell type-related genes, and genes involved in inflammation and EMT (**Table S3**).

**Figure 2:**
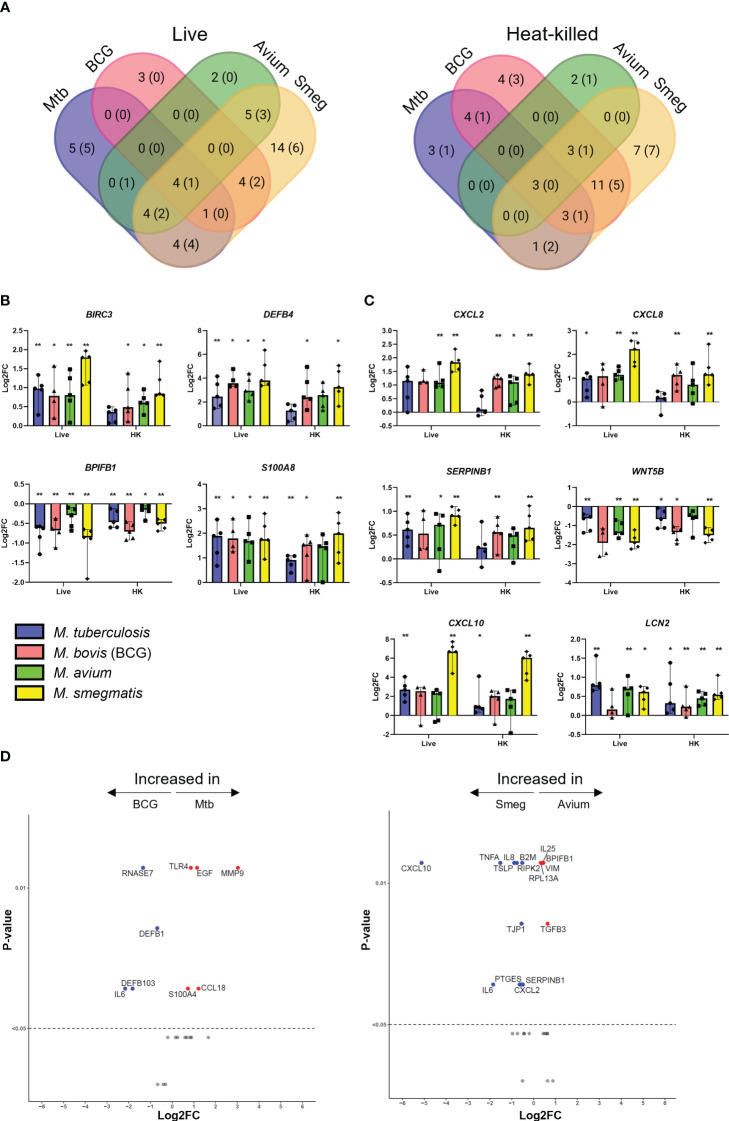
Mycobacterial infection promotes a shared gene expression signature in well-differentiated primary bronchial epithelial cell cultures. **(A)** Venn diagrams showing significantly differentially expressed genes (DEGs) uniquely induced or induced by multiple pathogens. DEGs determined with Mann-Whitney U test. Number indicates number of DEGs at p<0.05, with those at p<0.01 indicated in brackets. **(B)** Bar graphs showing significantly differentially regulated genes in common between ALI-PBEC infected with various mycobacteria. Statistics determined by Mann-Whitney U test. **(C)** Bar graphs depicting other highly up- or downregulated genes in ALI-PBEC in response to most mycobacterial species tested. Statistics determined by Mann-Whitney U test. **(D)** Volcano plots showing genes significantly differentially expressed in ALI-PBEC infected with Mtb versus BCG, and *M. avium* versus *M. smegmatis.* Data points above the dotted line are p < 0.05. DEGs determined by Mann-Whitney U test. P<0.05 is indicated by *. P<0.01 is indicated by **.

PBEC displayed transcriptomic changes at 48 h post infection by all mycobacterial strains. Four genes were consistently found to be significantly differentially expressed in PBEC infected with all four strains of live mycobacteria in comparison to uninfected cells (**Figure 2A**). *DEFB4, S100A8, and BIRC3* were significantly upregulated, while *BPIFB1* was significantly downregulated compared to uninfected control PBEC (**Figure 2B**). Expression of other genes that were strongly upregulated after infection with most mycobacteria included *SERPINB1, CXCL2, CXCL8, CXCL10*, and *LCN2* (**Figure 2C**). Expression of *WNT5B* on the other hand, was reduced in response to all tested mycobacteria, though this did not reach statistical significance for all species of mycobacteria (**Figure 2C**). Gene expression patterns in PBEC exposed to heat-killed bacteria followed largely the same pattern, albeit with smaller gene expression changes compared to live bacilli. Upon comparing live Mtb to live BCG (**Figure 2D**), the expression of several genes was increased by Mtb. These included *MMP9, S100A4, TLR4, EGF* and *CCL18.* On the other hand, BCG increased expression of *IL6, DEFB1, DEFB103*, and *RNASE7. A* similar comparative analysis of *M. avium* and *M. smegmatis* revealed that *M. smegmatis* increased several pro-inflammatory genes such as *CXCL10, IL6, CXCL8 and TNFA*, while *M. avium* increased genes related to cell adhesion such as *VIM* and *TGFB3.*

**Figure 3:**
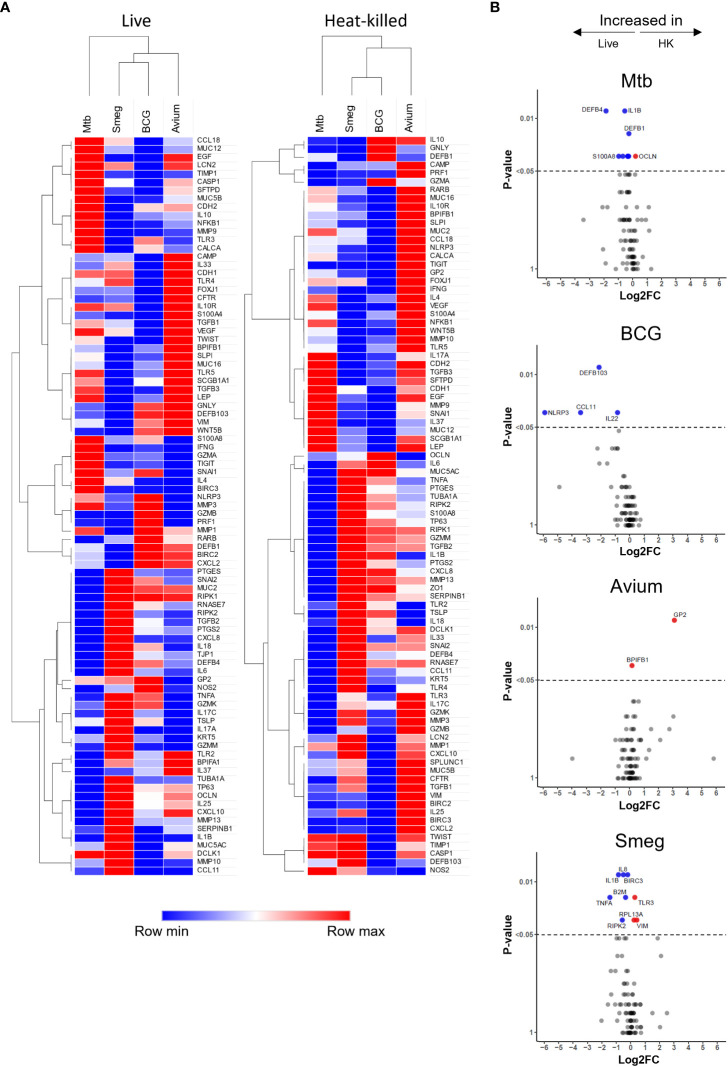
Gene expression profiles induced in ALI-PBEC infected with various live and heat-killed mycobacteria. **(A)** Heat maps of PBEC relative gene expression in response to live and heat-killed pathogens. Median log2 fold change to uninfected PBEC of 5 donor pools. The BCG Live sample group contains N=4 (instead of 5) different donor pools due to a technical issue leading to poor RNA yield from one donor pool. Clustering method used is one minus Spearman rank correlation. **(B)** Volcano plots showing genes significantly differentially expressed in ALI-PBEC upon infection with live versus heat-killed bacteria of each species. Data points above the dotted line are p < 0.05. DEGs determined by Mann-Whitney U test.

Hierarchical clustering revealed that both live bacteria and heat-killed bacteria induced gene expression changes in PBEC that were most similar for BCG and *M. smegmatis*, followed by *M. avium*, while Mtb-induced gene expression patterns were most different from those induced by the other strains (**Figure 3A**). Direct comparison of transcriptome changes induced by live and heat-killed bacteria of each pathogen revealed that more genes are significantly upregulated in response to live bacteria (**Figure 3B**). For Mtb, this included mostly antimicrobials such as *DEFB4* and *S100A8.* Interestingly, changes between live and heat-killed bacteria were different for each mycobacterium.

Of note, for many of the analysed genes, the magnitude of the epithelial response did not seem to be dependent on infection percentage. For example, induction of *S100A8* expression was nearly equal for all four mycobacteria (**Figure 2B**), while infection percentages between the four strains were variable (**Figure 1C**).

Besides the gene expression signature described above, pathogen-specific induction of gene expression was also observed. For example, expression of *MMP9* was increased by Mtb but strongly reduced by the other mycobacteria (**Figure S2**). Differences between pathogens were also detectable in the EMT regulatory factor *SNAI2. M. smegmatis* significantly upregulated this transcription factor, while the other species did not. Furthermore, several cell lineage gene markers were influenced by infection. Club cell marker *SCGB1A1* was significantly downregulated in epithelium by BCG and *M. smegmatis*, and Mtb and *M. avium* induced a trend similar to the other pathogens. Furthermore, expression of ciliated cell marker *TUBA1A* was significantly increased only by *M. smegmatis. DCLK1*, which is considered a marker for tuft cells, was slightly upregulated by all pathogens, though only for BCG this reached statistical significance.

To summarize, we have shown that the four species of mycobacteria induce a common gene expression pattern in PBEC, which consists mainly of antimicrobials and pro-inflammatory cytokines, though species-specific induction of differential gene expression was also observed.

### Secretion of a common cytokine profile by bronchial epithelial cultures in response to all mycobacteria

Epithelial cells secrete a plethora of cytokines and chemokines during homeostasis and upon infection, to interact with surrounding cells and tissue, including the immune system. We determined cytokine profiles of well-differentiated PBEC cultures in response to heat-killed and live mycobacteria. As ALI-PBEC cultures are polarized, samples were collected from both the apical (apical wash) and basal (medium) side.

**Figure 4:**
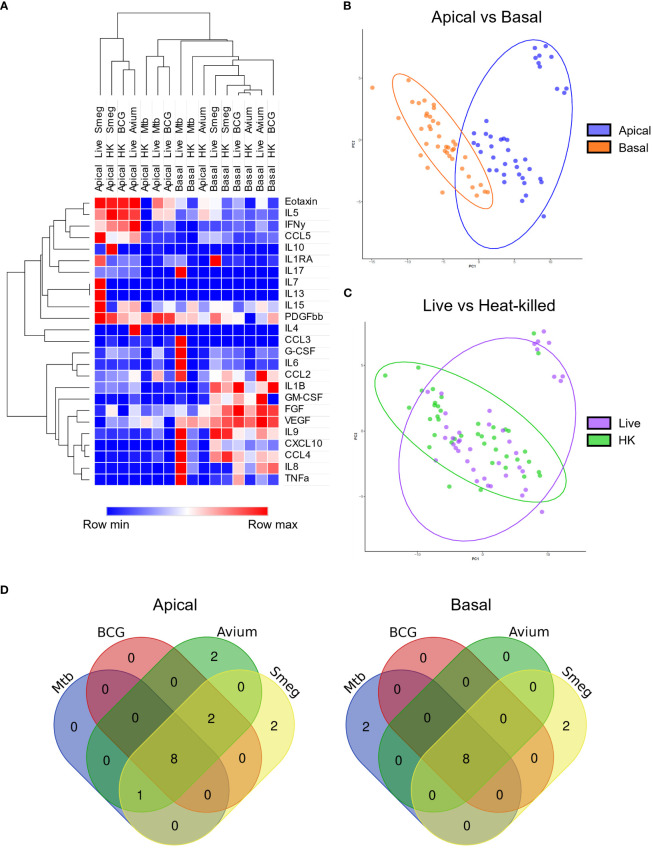
Polarized secretion of cytokine profiles by mycobacteria-infected well-differentiated primary bronchial epithelial cell cultures. **(A)** Heat map depicting changes in levels of apically and basally secreted cytokines by PBEC after infection with mycobacteria. Median fold change to secretion by uninfected PBEC of N = 5 different donor pools. Clustering method used is one minus Spearman rank correlation. **(B)** PCA performed on apical and basal samples. **(C)** PCA performed on live and heat-killed samples. **(D)** Venn diagrams depicting significantly differentially secreted cytokines unique per pathogen and overlapping between pathogens. Statistical significance was tested using a Mann-Whitney U test with FDR correction (Benjamini-Hochberg) for multiple testing.

First, secreted cytokine profiles were analysed by principal component analysis (PCA) and hierarchical clustering. Both methods separated apical and basal samples, indicating a polarized secretion of molecules (**Figures 4A, B**). In agreement with the similarity in patterns found in the gene expression analysis, also the cytokines secreted in response to live, or heat-killed mycobacteria did not seem to differ notably (**Figure 4C** and **Figure S3**). Clustering identified a group of inflammation-related cytokines secreted on the apical side in response to infection, including IL-5, IL-17, IL-10 and IL-1RA. In contrast, the basal production contained mainly tissue growth factors and chemokines, such as VEGF, FGF, GM-CSF, CXCL10 and CCL4 (**Figure 4A**).

All cytokines that were significantly differentially secreted in response to live infection were compiled and plotted as a Venn diagram (**Figure 4D**) to examine the common cytokine response to mycobacteria. The 8 cytokines induced by live bacteria of all four species in all different donor mixes are listed in **Tables 1**, **2**. Overlapping cytokines produced both apically and basally included chemotactic factors G-CSF, CCL2, CCL4 and CCL11. Furthermore, pro-inflammatory cytokines IL-6 and IL-9 were highly upregulated in response to all mycobacteria. Finally, apical secretion of IL-1β and IL-5, and basal secretion of CXCL10 and TNF-α were strongly upregulated in response to all mycobacteria.

**Table 1:** Apical cytokines induced by live bacteria of all four species of mycobacteria.

Apical cytokine		G-CSF		IL-6		IL-9	CCL2	CCL4	CCL11	IL-1β	IL-5
**Uninfected**	*Median*	775		456		392	81	3	3	93	554
	*Q1*	455		269		281	56	2	1	77	495
	*Q3*	1281		751		436	192	3	4	109	619
	*IQR*	827		481		155	136	1	3	31	124
***M. tuberculosis***	*Median*	17295		3709		588	1289	8	14	142	1048
	*FC*	22,3		8,1		1,5	15,8	2,9	4,4	1,5	1,9
	*Pools +*	5/5		5/5		5/5	5/5	5/5	5/5	5/5	5/5
	*Q1*	16698		3677		576	1026	8	10	136	1014
	*Q3*	17705		4183		598	1984	10	24	152	1062
	*IQR*	1007		506		22	958	3	15	15	48
***M. bovis* (BCG)**	*Median*	12377		4620		564	1739	8	18	141	1048
	*FC*	16,0		10,1		1,4	21,4	2,6	5,7	1,5	1,9
	*Pools +*	5/5		5/5		5/5	5/5	5/5	5/5	5/5	5/5
	*Q1*	12119		3092		560	1517	8	12	133	913
	*Q3*	16276		4697		590	1932	8	22	144	1213
	*IQR*	4157		1605		30	415	0	10	11	300
***M. avium***	*Median*	30196		5813		653	2982	12	24	156	1474
	*FC*	39,0		12,7		1,7	36,6	4,2	7,4	1,7	2,7
	*Pools +*	5/5		5/5		5/5	5/5	5/5	5/5	5/5	5/5
	*Q1*	25813		3831		640	2437	8	22	152	1181
	*Q3*	31766		6873		746	3002	13	26	170	1508
	*IQR*	5952		3042		106	565	4	4	18	327
***M. smegmatis***	*Median*	16375		5832		649	1501	11	21	145	1247
	*FC*	21,1		12,8		1,7	18,4	3,7	6,5	1,6	2,2
	*Pools +*	5/5		5/5		5/5	5/5	5/5	5/5	5/5	5/5
	*Q1*	14948		5260		643	1497	8	20	141	1214
	*Q3*	17652		7911		748	1594	11	34	155	1540
	*IQR*	2704		2651		106	97	3	14	14	326

Data represented as median pg/ml with interquartile range (IQR) and fold change (FC) to uninfected. The number of donor pools in which a cytokine was differentially secreted upon infection (Pools +) is given as x out of 5 pools (e.g. 5/5).

All apical IL-8 samples were over the detection limit. Therefore IL-8 is not included in these results.

**Table 2:** Basal cytokines induced by live bacteria of all four species of mycobacteria.

Basal cytokine		G-CSF	IL-6		IL-9	CCL2	CCL11	TNFα	CXCL10	CCL4
**Uninfected**	*Median*	766	97		553	119	8	1	1	163
	*Q1*	576	69		509	108	8	1	1	155
	*Q3*	1031	139		623	136	11	1	1	190
	*IQR*	455	71		114	28	3	0	0	35
***M. tuberculosis***	*Median*	39757	2875		2082	3224	24	1471	1726	572
	*FC*	51,9	29,7		3,8	27,2	2,9	1471,0	1726,1	3,5
	*Pools +*	5/5	5/5		5/5	5/5	5/5	5/5	5/5	5/5
	*Q1*	36108	2718		2062	1532	21	790	1295	548
	*Q3*	46299	3926		2431	3251	24	1613	1741	727
	*IQR*	10191	1208		368	1720	2	823	446	178
***M. bovis* (BCG)**	*Median*	21484	1508		1833	2128	16	998	703	539
	*FC*	28,0	15,6		3,3	17,9	2,0	998,0	702,9	3,3
	*Pools +*	5/5	5/5		5/5	5/5	5/5	5/5	5/5	5/5
	*Q1*	9399	684		1803	978	16	349	661	466
	*Q3*	25322	2442		1913	2434	20	1394	762	554
	*IQR*	15922	1758		110	1457	4	1045	101	88
***M. avium***	*Median*	15526	1170		1973	3821	16	464	572	526
	*FC*	20,3	12,1		3,6	32,2	2,0	464,0	572,1	3,2
	*Pools +*	5/5	5/5		5/5	5/5	5/5	5/5	5/5	5/5
	*Q1*	13850	1100		1943	2117	14	464	548	507
	*Q3*	19803	1369		2022	4636	19	722	703	557
	*IQR*	5953	269		80	2518	4	258	155	50
***M. smegmatis***	*Median*	14922	2004		2212	1233	19	327	1025	566
	*FC*	19,5	20,7		4,0	10,4	2,3	326,9	1024,8	3,5
	*Pools +*	5/5	5/5		5/5	5/5	5/5	5/5	5/5	5/5
	*Q1*	10881	1459		2032	1023	16	154	855	519
	*Q3*	24902	3792		2617	2426	20	722	1149	603
	*IQR*	14021	2333		585	1403	4	567	294	85

Data represented as median pg/ml with interquartile range (IQR) and fold change (FC) to uninfected. The number of donor pools in which a cytokine was significantly differentially secreted upon infection (Pools +) is given as x out of 5 pools (e.g. 5/5).

In conclusion, we illustrated that mycobacterial infection induced secretion of a polarized common cytokine profile by PBEC, which consisted of pro-inflammatory cytokines apically and growth factors and chemokines basally.

### Production of mycobacterial species-specific cytokine profiles by PBEC cultures

**Figure 5:**
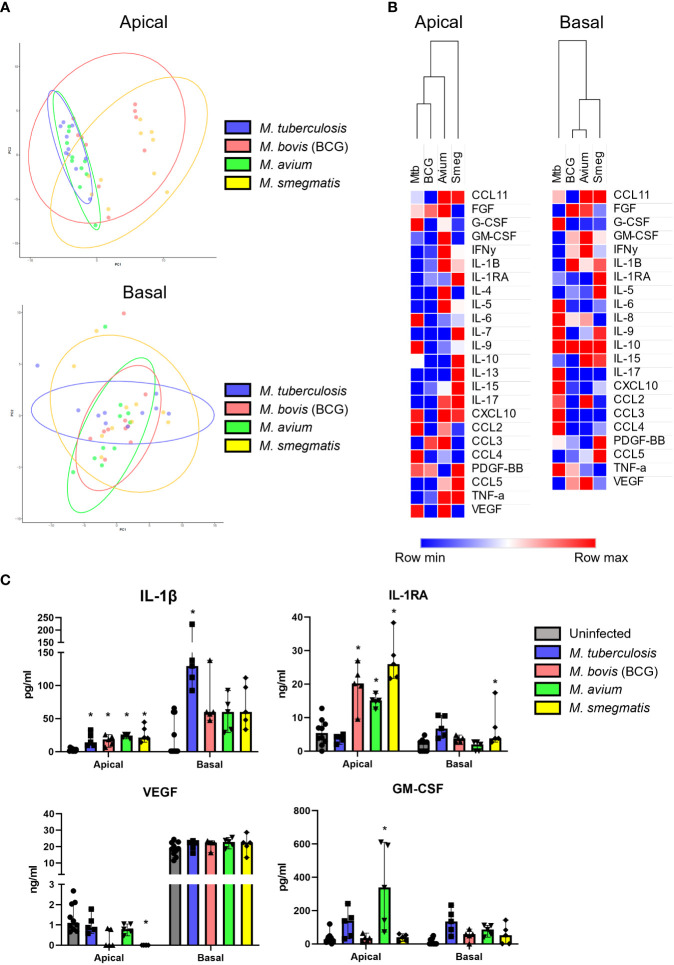
Mycobacterial species-specific cytokine profiles produced by well-differentiated bronchial epithelial cell cultures. **(A)** PCAs comparing pathogen-specific responses, for apical and basal samples. **(B)** Heat maps depicting changes in apically and basally secreted cytokines by well-differentiated primary bronchial epithelial cell cultures after infection with mycobacteria. Median fold change to secretion by uninfected PBEC of N = 5 different donor pools. **(C)** Cytokines with patterns unique to single pathogens. Mann-Whitney U test with FDR correction (Benjamini-Hochberg) for multiple testing. P<0.05 is indicated by *.

To investigate whether mycobacteria also induce cytokine profiles that are species-specific in addition to the common profile described above, PCA was performed (**Figure 5A**). Even though epithelial cytokine production induced by the pathogens showed considerable overlap with each other (see above), on the apical side Mtb and *M. avium* were displaying more similarities to each other than to the other species in their induced cytokine expression profile. BCG and *M. smegmatis-*induced cytokine profiles also grouped together on the basis of being more similar to each other than to other pathogens. On the basal side, however, the four pathogens induced profiles that were similar. Clustering analysis of apical secretions showed that Mtb- and BCG-induced cytokine profiles were more comparable (**Figure 5B**), contradicting results found with PCA analysis.

Detailed analysis of the subtle differences between mycobacterial strains that were observed by PCA showed that apical IL-1β release was significantly induced by all pathogens apically, whereas only Mtb significantly triggered its basal release (**Figure 5C**). In contrast, Mtb-infected PBEC secreted similar levels of IL-1RA as uninfected cells, whereas the other mycobacteria significantly upregulated apical IL-1RA secretion. Finally, *M. smegmatis* infection significantly downregulated VEGF secretion apically, while levels secreted upon infection with Mtb and *M. avium* remained similar to those of uninfected PBEC. Three out of five BCG-infected PBEC pools showed drastic downregulation of VEGF as well, but this did not reach statistical significance. Finally, GM-CSF was significantly upregulated in *M. avium*-infected PBEC and not by other species, albeit only apically.

In short, we have established that infection of PBEC by different mycobacterial species induced different up- and downregulation of several cytokines.

### Cytokines secreted by infected ALI-PBEC attract neutrophils

Cytokines secreted by epithelial cells may act as chemoattractants for various immune cell subsets. To assess whether secretions from infected ALI-PBEC could attract neutrophils, which are generally first responders during bacterial infections, a neutrophil migration assay was performed. Basal medium and apical supernatants from five infected ALI-PBEC donor mixes per mycobacterial species were pooled (**Supplementary Figure S4A**), and used to attract neutrophils isolated from three independent healthy donors. N-formyl-met-leu-phe (fMLP) with 10% fetal bovine serum (FBS) which induces chemotaxis and degranulation of neutrophils, was used as a positive control. The number of migrated neutrophils was determined by flow cytometry (gating strategy depicted in **Supplementary Figure S4B**).

**Figure 6:**
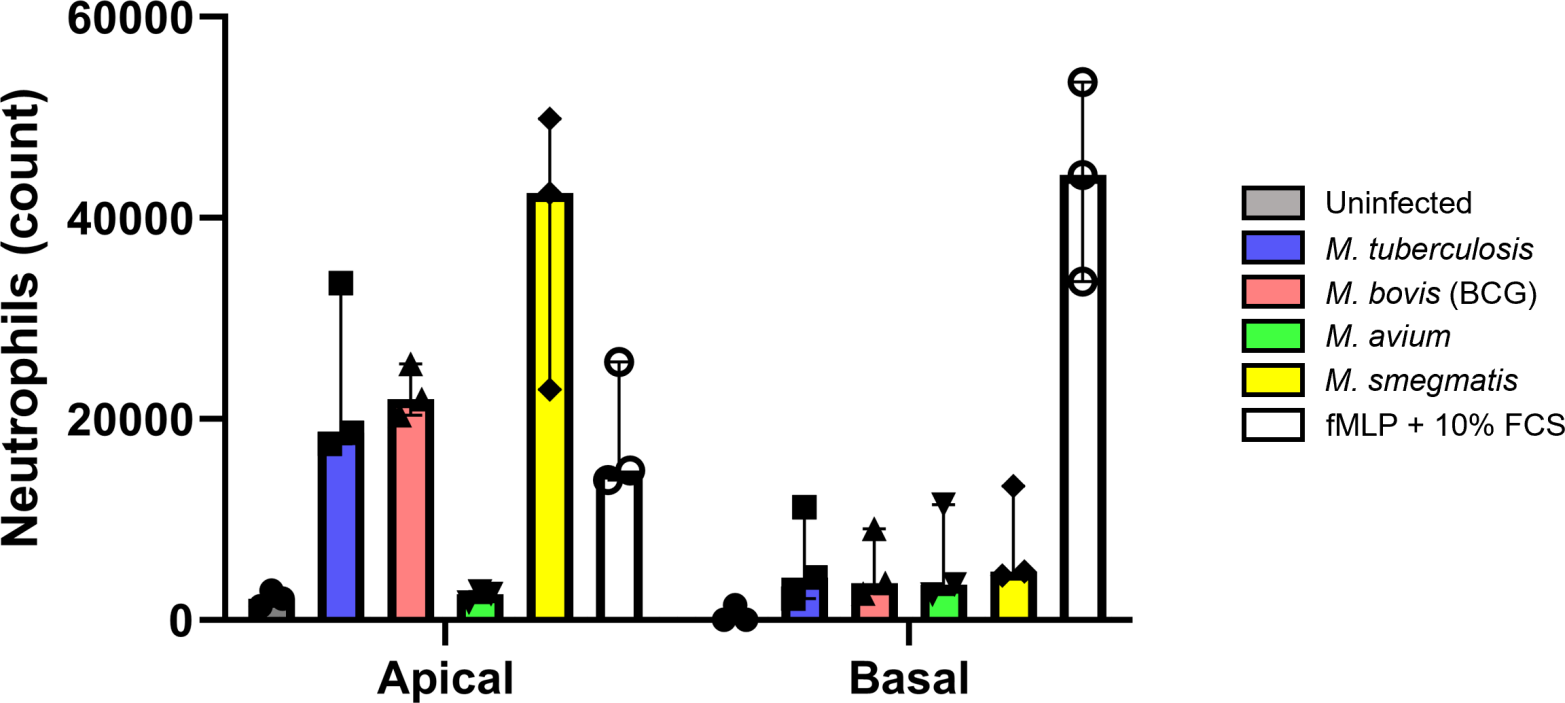
Cytokines secreted by infected well-differentiated bronchial epithelial cell cultures are chemotactic for neutrophils. Bar graph depicting neutrophil counts after migration upon exposure to apical wash or basal cell culture medium derived from infected air-liquid interface cultures of primary bronchial epithelial cells. Cell numbers are corrected for spontaneous migration to PBS or cell culture medium. Data points represent N=3 different healthy neutrophil donors.

Firstly, a much higher number of neutrophils migrated in response to apical wash compared to basal medium. This difference is evident in the increased chemotactic capacity of the respective uninfected control. In addition, the number of neutrophils attracted by apical secretions of Mtb, BCG and *M. smegmatis* even surpassed those attracted by the fMLP + 10% FBS control. Although apical wash was 2.5 times more concentrated than basal medium, apical wash from infected PBEC attracted approximately 4 times as many neutrophils compared to basal medium (**Figure 6**). This was true for both the uninfected as well as infected conditions, with exception of *M. avium* basal medium. Furthermore, we observed several differences between the four species. Apical secretions of Mtb and BCG-infected ALI-PBEC attracted neutrophils to a similar extent as fMLP with 10% FBS, while secretions from *M. smegmatis*-infected ALI-PBEC even surpassed those numbers. Surprisingly, apical secretions from cells infected with *M. avium* barely attracted any neutrophils. In fact, those numbers were close to the number of neutrophils that spontaneously migrated towards uninfected ALI-PBEC secretions. On the other hand, the numbers of neutrophils attracted by basal secretions from infected ALI-PBEC were very similar across all pathogens, though still higher than the uninfected condition.

Taken together, cytokines secreted by PBEC infected with mycobacteria, except *M. avium*, were able to attract neutrophils from healthy independent donors.

## Discussion

In the lung, epithelial cells not only serve as a barrier to the outside world, but are also the first cells to encounter airborne pathogens. Here, we show that four different mycobacterial species, namely *M. tuberculosis, M. bovis* (BCG), *M. avium and M. smegmatis*, can directly infect primary human bronchial epithelial cells with varying but generally low efficiency. A common epithelial response to mycobacterial infection consisted of upregulation of *BIRC3, S100A8* and *DEFB4*, and downregulation of *BPIFB1* gene expression. In addition, mediator secretion was polarized and upon infection revealed a skewing towards inflammation-related cytokines on the apical side of the cultures, and towards chemotactic cytokines and growth factors on the basal side of the cultures. Secreted cytokines were largely overlapping between all four species and included G-CSF, CCL11, CCL2, IL-6, and IL-9, that were secreted on both sides, in addition to apical secretion of CCL4 and IL-5, and basal secretion of CXCL10 and IL-8. To our knowledge, bronchial epithelial host responses, particularly from primary cells, to a range of mycobacteria has not been previously described in this level of detail.

A limitation of our study is the use of supraphysiological concentrations of bacteria. Despite using relatively high MOIs, mycobacterial infection of PBEC seems less efficient compared to infection percentages of monocyte-derived macrophages achieved by other studies from our group (Kilinc et al., 2022). This could indicate the presence of stronger defence mechanisms of airway epithelium against mycobacteria and/or less efficient phagocytosis of the bacteria. A previous study using Mtb H37Rv to infect PBEC found that CFU retrieved from infected PBEC were 2 log-fold lower than those from THP-1 macrophages (Reuschl et al., 2017). Our CFU data correspond well with those findings. A different study using *M. avium at* MOI 100 to infect PBEC found a higher infection percentage of approximately 25% (Matsuyama et al., 2018), compared to the infection rate of 11% we found for *M. avium.* However, this does not approach the infection rates of *M. avium in* monocyte-derived macrophages, which reached between 60-90% at MOI 100 (Kilinc et al., 2022).

We have used mixes of 4 donors per mix to generate ALI-PBEC cultures, instead of single donors. Since the donor mixes minimize inter-donor variability, we do not capture donor-specific effects in this study, but rather general host responses that may represent a broader population. While Mtb and BCG infection rates appeared steady over different mixes, there was considerable variation in infection rates observed for *M. avium and M. smegmatis*. It will be interesting to further delineate what donor (culture) characteristics are responsible for these changes, for example cellular composition, sex differences, or mucus composition (both secreted and membrane-bound mucins) which could be factors involved.

The overlapping gene expression profile in mycobacteria-infected well-differentiated PBEC cultures included antimicrobials such as *DEFB4, S100A8* and *BPIFB1.* Human beta-defensin 2 (hBD2) is encoded by *DEFB4*, and is expressed by a variety of cells, including the alveolar epithelial cell line A549. Upon infection of these cells with Mtb, hBD2 was demonstrated to be present on Mtb membranes (Rivas-Santiago et al., 2005). Its expression was also demonstrated in Mtb-infected airway organoids (Iakobachvili et al., 2022). *S100A8* encodes one half of calprotectin heterodimer, which has previously been shown to facilitate neutrophil accumulation in mice. *S100A8* expression is also increased in whole blood of human TB progressors versus non-progressors (Scott et al., 2020). In a different study, calprotectin levels in plasma of TB patients were also significantly increased compared to healthy controls and pulmonary sarcoidosis patients. In liquid medium cultures, S100A8/A9 complex significantly increased Mtb growth in a dose- and time-dependent manner (Pechkovsky et al., 2000), though the exact mechanism underlying this effect remains unknown. *BPIFB1* encodes the antimicrobial LPLUNC, and its downregulation has, to our knowledge, not been associated with mycobacterial infection previously. PLUNC proteins are highly expressed by secretory epithelial cells, such as club cells and goblet cells (Bingle and Bingle, 2011). Therefore, besides ordinary downregulation of the gene, a reduction in *BPIFB1* mRNA could indicate a decrease in these cell types. Additional research on cell type ratios following mycobacterial infection would be necessary to confirm this hypothesis. Although not statistically significant, *LCN2* was also highly upregulated. *LCN2* encodes lipocalin-2, which may regulate inflammation during mycobacterial infections and thereby constrain T cell lymphocytic accumulation (Guglani et al., 2012). Furthermore, *LCN2-*deficient mice are highly susceptible to intratracheal infection with Mtb (Saiga et al., 2008). Regulation of antimicrobials could indicate a direct role for the epithelium in clearance (or inhibition thereof) of mycobacteria. The final gene found significantly upregulated by all mycobacteria was *BIRC3*, which encodes a member of the IAP family of proteins that regulates NFκB signalling and inhibits apoptosis (Gyrd-Hansen and Meier, 2010; Estornes and Bertrand, 2015). This could indicate a mechanism to increase resistance to cell death by PBEC upon infection with mycobacteria.

The comparison of transcriptomes induced by live and heat-killed Mtb and BCG may identify factors related to Mtb virulence. When comparing live Mtb and BCG, *MMP9* was increased by Mtb. It was previously shown that *MMP9* expression is induced in lung epithelium by ESAT-6, a secreted Mtb protein (Volkman et al., 2010). As ESAT-6 is absent in BCG, and *MMP9* expression is not increased by BCG, our results confirmed that this is an Mtb-specific effect. However, upon comparing live with heat-killed Mtb, no significant difference was found with regards to *MMP9.* Instead, there seemed to be an increase of antimicrobial gene expression when ALI-PBEC were stimulated with live bacteria. Furthermore, the expression pattern induced by heat-killed Mtb is not completely similar to the pattern induced by live BCG, even in the small selection of genes tested in this study, which may be relevant in the context of vaccine development.

Cytokines secreted highly on both the apical and basal side of infected ALI-PBEC cultures included factors that might be essential for initiating an immune response to mycobacteria. For example, apical secretion of CCL4 and IL-5 was increased compared to uninfected controls. CCL4 was previously demonstrated to suppress intracellular growth of Mtb in macrophages (Saukkonen et al., 2002), and IL-5 promotes maturation, survival and chemotaxis of eosinophils and induces B cell immunoglobulin production and isotype switching (Purkerson and Isacson, 1992; Takatsu and Nakajima, 2008). Therefore, despite being relatively resistant to mycobacterial infection, the bronchial epithelium may play a pivotal role not only as an early warning signal for immune cells, but also to support immune cell recruitment and differentiation in response to the infection.

In both our cytokine and gene expression data, we observed a large overlap in epithelial responses to heat-killed or live bacteria, although responses to heat-killed bacteria were less strong than responses to live bacteria. We did, however, observe a striking difference in secretion of IL-6 and CCL2, as well as IL-8 and IL-17 (basal only) when PBEC were stimulated with heat-killed Mtb compared to live Mtb. These differences were less pronounced or absent when comparing heat-killed and live versions of the other pathogens. Interestingly, CXCL10 and TNF-α secretion remained stable in all basal secretions and in most apical secretions, regardless of whether the pathogens were live or heat-killed. These findings indicate that these cytokines may be triggered by a heat-stable component. Monocytes derived from TB patients and healthy controls stimulated with heat-killed Mtb were shown to produce significant amounts of TNF-α (Selenscig et al., 2009). In another study, mice receiving ocular intravitreal injection with heat-killed Mtb produced CXCL10 (Pepple et al., 2022). The authors also found that CCL2 was not produced by these mice unless they had been previously primed with a subcutaneous injection of heat-killed Mtb. IL-17 was produced by mice receiving heat-killed Mtb, though in low concentrations.

Neutrophils migrated towards secretions of infected ALI-PBEC, showing that in response to mycobacteria, epithelial cells secrete effector molecules that can attract immune cells. Interestingly, apical secretions from PBEC exposed to Mtb, BCG and *M. smegmatis* were highly chemotactic to neutrophils whereas secretions from those exposed to *M. avium* were not. In apical secretions of *M. avium*-infected PBEC, we detected a significantly higher concentration of GM-CSF compared to the other pathogens and uninfected PBEC. GM-CSF was produced at low levels by uninfected PBEC, and production was significantly increased by approximately 12-fold in response to *M. avium* infection. GM-CSF levels of Mtb-infected PBEC were elevated approximately 5-fold compared to uninfected PBEC, though this did not reach statistical significance. Previous studies have demonstrated that short pre-incubation of neutrophils with GM-CSF significantly reduced their migration capacity (Kharazmi et al., 1988; Castellani et al., 2019). We hypothesize that the higher amount of GM-CSF in *M. avium*-induced cytokine secretions from PBEC potentially inhibited neutrophil migration.

In light of our findings, it will be important to characterize epithelial responses to such mucosal vaccines as well since epithelial cells may have the capacity to orchestrate and direct initial immune responses to Mtb. The BCG vaccine is currently the only effective vaccine against TB, but its protective efficacy against pulmonary TB is variable. The vaccine is administered via the intradermal route, and induces a strong systemic response, but weak mucosal responses. In attempts to improve the efficacy of BCG, various studies have tried mimicking the route of infection by mucosal vaccination (Stylianou et al., 2019). In mouse models of TB, mucosal vaccination conferred superior protection compared to the systemic intradermal route, and resulted in an increased frequency of CD4^+^ antigen-specific T cells (Bull et al., 2019). In a different study, intratracheal and intranasal BCG vaccination of mice generated an increased number of T effector memory cells and tissue-resident memory T cells (Perdomo et al., 2016). In rhesus macaques, mucosal BCG vaccination also conferred strongly enhanced protection to Mtb infection compared to standard intradermal vaccination (Dijkman et al., 2019). In addition, mucosal but not intradermal vaccination of rhesus macaques with BCG or the Mtb-derived vaccine candidate MTBVAC enhanced cytokine production by monocytes. This was associated with metabolic rewiring and is a typical trait of trained immunity (Vierboom et al., 2021). These studies have focused mainly on delineating the immune responses following vaccination.

In conclusion, we report that primary bronchial epithelial cells can be directly infected by four species of mycobacteria. Infection was associated with a common host response consisting mainly of enhanced expression of antimicrobial genes, and increased secretion of cytokines and chemokines that showed chemotactic activity towards neutrophils. These findings highlight the likely involvement of the airway epithelial compartment in the initial host-pathogen interactions in TB and related NTM, and provide novel insights into the earliest stages of human immunity to mycobacteria.

## Data availability statement

The original contributions presented in the study are included in the article/**Supplementary Materials**, further inquiries can be directed to the corresponding author/s.

## Ethics statement

Ethical approval was not required for the studies involving humans because patients from which lung tissue was derived were enrolled in the biobank via a no-objection system for coded anonymous further use of such tissue (www.coreon.org). However, since 01-09-2022, patients are enrolled in the biobank using active informed consent in accordance with local regulations from the LUMC biobank with approval by the institutional medical ethical committee (B20.042/Ab/ab and B20.042/Kb/kb). The studies were conducted in accordance with the local legislation and institutional requirements. The human samples used in this study were acquired from a by- product of routine care or industry. 

## Author contributions

AB collected and analyzed data and drafted the manuscript. DN performed all cell culture work to create the ALI-PBEC *in vitro* models. SV performed large parts of the analysis of multiplex qPCR and Luminex data. PH, TO, AD, and SJ served as scientific advisors and critically reviewed the study design and manuscript. All authors contributed to the article and approved the submitted version.

## Supplementary material

Supplementary Table 1 Bacterial species used in this study.

Supplementary Table 2 Antibodies used for confocal microscopy.

Supplementary Table 3 Sequences of primer pairs used in Fluidigm gene expression assay.

Supplementary Figure 1 Flow cytometry gating strategies and comparison between flow cytometers. **(A)** Flow cytometry gating strategy to determine the percentage of mycobacteria-infected cells in the ALI-PBEC cultures. **(B)** Representative result of one flow cytometry experiment measuring the same samples on two different cytometers. **(C, D)** Comparison of percentage of infected cell in ALI-PBEC cultures infected with MOI 10 and 100 (N=3 independent experiments with different donor pools) between two cytometers. **(E, F)** Percentage infected cells of ALI-PBEC cultures infected with various mycobacterial species at MOI 10 and 100. Data are depicted per pathogen and per donor pool (n=5).

Supplementary Figure 2 Pathogen-specific differential gene expression patterns induced by mycobacterial infection. Bar graphs depicting pathogen-specific induction of differential gene expression in ALI-PBEC cultures infected with four mycobacterial species. Statistical significance determined by Mann-Whitney U test.

Supplementary Figure 3 Comparison of cytokine secretions induced by live or heat-killed mycobacteria in infected primary bronchial epithelial cells. **(A)** Heat maps depicting apically secreted cytokines by PBEC after infection with live and heat-killed mycobacteria. Changes in secretion were determined by comparing the median fold change to secretion by uninfected PBEC of 5 donor pools. **(B)** Heat maps depicting basally secreted cytokines by PBEC after infection with live and heat-killed mycobacteria. Changes in secretion were determined by comparing the median fold change to secretion by uninfected PBEC of 5 donor pools.

Supplementary Figure 4 Sample pooling strategy and flow cytometry gating strategy for neutrophil migration assay. **(A)** Schematic showing sample pooling strategy for apical wash and basal medium samples from infected ALI-PBEC. Pooled samples were used in the neutrophil migration assay. Created with BioRender.com. **(B)** Flow cytometry gating strategy to determine counts of neutrophils that migrated in response to apical wash and basal medium samples from infected ALI-PBEC.
