# Supplementary material for: Airway epithelial cells mount an early response to mycobacterial infection
Source: Front Cell Infect Microbiol. 2023 Sep 26;13:1253037. doi: 10.3389/fcimb.2023.1253037 (PMC10562574; doi:10.3389/fcimb.2023.1253037)
Supplement: Supplementary Table 1 — Bacterial species used in this study. [file Table_1.docx]

Table S1. Bacterial species used in this study

| **Bacterial species** | **Strain** | **Fluorescent tag** | **Plasmid** | **Source** |
| --- | --- | --- | --- | --- |
| *Mycobacterium avium* | ATCC 700898 | Wasabi | pSMT3-Wasabi | Fluorescent strain generated at own lab |
| *Mycobacterium bovis* | BCG P3 | GFP | pSMT3eGFP | Fluorescent strain generated at own lab |
| *Mycobacterium smegmatis* | mc^2^155 | GFP | pSMT3eGFP | Fluorescent strain generated at own lab |
| *Mycobacterium tuberculosis* | H37Rv mc^2^8120 | Venus | pYUB2133-Venus | Jacobs Lab, Bronx NY  USA |
