# Supplementary material for: Airway epithelial cells mount an early response to mycobacterial infection
Source: Front Cell Infect Microbiol. 2023 Sep 26;13:1253037. doi: 10.3389/fcimb.2023.1253037 (PMC10562574; doi:10.3389/fcimb.2023.1253037)
Supplement: Supplementary Table 2 — Antibodies used for confocal microscopy. [file Table_2.docx]

Table S2. Antibodies used for confocal microscopy

| **Target or name** | **Marker for** | **Host species** | **Target species** | **Concentration** | **Source** | **Cat. nr.** |
| --- | --- | --- | --- | --- | --- | --- |
| EpCAM | Epithelial cells | Goat | Human | 10 ug/ml | R&D Systems | AF960 |
| AlexaFluor 405 | Secondary Ab | Donkey | Goat | 1:200 | Invitrogen | A48259 |
