# Supplementary material for: Airway epithelial cells mount an early response to mycobacterial infection
Source: Front Cell Infect Microbiol. 2023 Sep 26;13:1253037. doi: 10.3389/fcimb.2023.1253037 (PMC10562574; doi:10.3389/fcimb.2023.1253037)
Supplement: Supplementary Table 3 — Sequences of primer pairs used in Fluidigm gene expression assay. [file Table_3.docx]

Table S3. Sequences of primer pairs used in Fluidigm gene expression assay.

| **Target** | **Forward primer 5’ 🡪 3’** | **Reverse primer 5’ 🡪 3’** |
| --- | --- | --- |
| *ATP5B* | TCACCCAGGCTGGTTCAGA | AGTGGCCAGGGTAGGCTGAT |
| *B2M* | GACCACTTACGTTCATTGACTCC | CAGGGTTTCATCATACAGCCAT |
| *BIRC2* | AGCACGATCTTGTCAGATTGG | GGCGGGGAAAGTTGAATATGTA |
| *BIRC3* | GCCATTGACTTTTCTGTCGCC | GCAAAGCAAGCCACTCTGT |
| *BPIFA1* | CTTGGCCTTGTGCAGAGC | CAACAGACTTGCACCGACC |
| *BPIFB1* | CAGTGCCATGCGGGAAAAG | GCTGGAGGATGTTAGCTGTGA |
| *CALCA* | TCTAAGCGGTGCGGTAATCTG | CAGTTTGGGGGAACGTGTGA |
| *CAMP* | TCATTGCCCAGGTCCTCAG | TCCCCATACACCGCTTCAC |
| *CASP1* | TTTCCGCAAGGTTCGATTTTCA | GGCATCTGCGCTCTACCATC |
| *CCL11* | GAAACCACCACCTCTCACG | GCTCTCTAGTCGCTGAAGGG |
| *CCL18* | AAGGAGGCCAGGAGTTGTGA | GGAGGTATAGACGAGGCAGC |
| *CDH1* | TTCCCAACTCCTCTCCTG | AAACCTTGCCTTCTTTGTC |
| *CDH2* | AGCCAACCTTAACTGAGGAGT | GGCAAGTTGATTGGAGGGATG |
| *CFTR* | TGCCCTTCGGCGATGTTTTT | GTTATCCGGGTCATAGGAAGCTA |
| *CXCL10* | GTGGCATTCAAGGAGTACCTC | TGATGGCCTTCGATTCTGGATT |
| *CXCL2* | GGCAGAAAGCTTGTCTCAACCC | CTCCTTCAGGAACAGCCACCAA |
| *CXCL8* | CTGGACCCCAAGGAAAAC | TGGCAACCCTACAACAGAC |
| *DCLK1* | CCAACTGGTCGGTGAACGTC | CTTGGGCCGAATGAAATCCTTA |
| *DEFB1* | ATGAGAACTTCCTACCTTCTGCT | TCTGTAACAGGTGCCTTGAATTT |
| *DEFB103* | TCCAGGTCATGGAGGAATCAT | CGAGCACTTGCCGATCTGT |
| *DEFB4* | ATCAGCCATGAGGGTCTTG | GCAGCATTTTGTTCCAGG |
| *EGF* | TGCAGAGGGATACGCCCTAA | CAAGAGTACAGCCATGATTCCAAA |
| *FOXJ1* | GGAGGGGACGTAAATCCCTA | TTGGTCCCAGTAGTTCCAGC |
| *GNLY* | CCTGTCTGACGATAGTCCAAAAA | GACCTCCCCGTCCTACACA |
| *GP2* | GGAGAGTTCATTGTCAGGATGG | GCACGGACTCAACAGACAGTT |
| *GZMA* | TCTCTCTCAGTTGTCGTTTCTCT | GCAGTCAACACCCAGTCTTTTG |
| *GZMB* | CCCTGGGAAAACACTCACACA | GCACAACTCAATGGTACTGTCG |
| *GZMK* | GGTGTTCTGATTGATCCACAGT | TGTGCGCCTAAAACCACAGT |
| *GZMM* | ACACCCGCATGTGTAACAACA | GGAGGCTTGAAGATGTCAGTG |
| *IFNG* | TCGGTAACTGACTTGAATGTCCA | TCGCTTCCCTGTTTTAGCTGC |
| *IL10* | GACTTTAAGGGTTACCTGGGTTG | TCACATGCGCCTTGATGTCTG |
| *IL10RA* | ATGAGCATTCAGACTGGGTAAAC | TTTTAGGGGCTAAGAAACGCAT |
| *IL17A* | TCCCACGAAATCCAGGATGC | GGATGTTCAGGTTGACCATCAC |
| *IL17C* | CCACACTGCTACTCGGCTG | CACACGGTATCTCCAGGGTGA |
| *IL18* | TCTTCATTGACCAAGGAAATCGG | TCCGGGGTGCATTATCTCTAC |
| *IL1B* | ATGATGGCTTATTACAGTGGCAA | GTCGGAGATTCGTAGCTGGA |
| *IL22* | GCTTGACAAGTCCAACTTCCA | GCTCACTCATACTGACTCCGT |
| *IL25* | AAGGAGATGGTTGGTCAGAAGA | CTCCTAATCGCAAAAGAGCATC |
| *IL33* | ACTCACTGTCACATTGGGCA | GCATCCAGAGGTCAGGTGAT |
| *IL37* | TGAACCCCAGTGCTGCTTAG | AGCCCACCTGAGCCCTATAA |
| *IL4* | CCAACTGCTTCCCCCTCTG | TCTGTTACGGTCAACTCGGTG |
| *IL6* | CAGAGCTGTGCAGATGAGTACA | GATGAGTTGTCATGTCCTGCA |
| *KRT5* | AGGAGTTGGACCAGTCAACAT | TGGAGTAGTAGCTTCCACTGC |
| *LCN2* | CCTCAGACCTGATCCCAGC | CAGGACGGAGGTGACATTGTA |
| *LEP* | AATGCATTGGGGAACCCTGT | AGGAGACTGACTGCGTGTGT |
| *MMP1* | AAAATTACACGCCAGATTTGCC | GGTGTGACATTACTCCAGAGTTG |
| *MMP10* | TGCTCTGCCTATCCTCTGAGT | TCACATCCTTTTCGAGGTTGTAG |
| *MMP13* | CCAGACTTCACGATGGCATTG | GGCATCTCCTCCATAATTTGGC |
| *MMP3* | CTGGACTCCGACACTCTGGA | CAGGAAAGGTTCTGAAGTGACC |
| *MMP9* | ACCTCGAACTTTGACAGCGAC | GAGGAATGATCTAAGCCCAGC |
| *MUC12* | CCAGTTCAAGCGACCCTTTTA | CGCTGTGGGATACTGTTGATT |
| *MUC16* | GGAGCACACGCTAGTTCAGAA | GGTCTCTATTGAGGGGAAGGT |
| *MUC2* | GACGCACTGTATCATCAAACG | AGGATGGTCGTGTTGATGCG |
| *MUC5AC* | CCTTCGACGGACAGAGCTAC | TCTCGGTGACAACACGAAAG |
| *MUC5B* | GCCTACGAGGACTTCAACGTC | CCTTGATGACAACACGGGTGA |
| *NFKB* | GCTTGTAGGAAAGGACTGCC | GTTGTTGTTGGTCTGGATGC |
| *NLRP3* | GATCTTCGCTGCGATCAACAG | CGTGCATTATCTGAACCCCAC |
| *NOS2* | TTCAGTATCACAACCTCAGCAAG | TGGACCTGCAAGTTAAAATCCC |
| *OCLN* | AAGAGTTGACAGTCCCATGGCATAC | ATCCACAGGCGAAGTTAATGGAAG |
| *PRF1* | GGCTGGACGTGACTCCTAAG | CTGGGTGGAGGCGTTGAAG |
| *PTGES* | TCCTAACCCTTTTGTCGCCTG | CGCTTCCCAGAGGATCTGC |
| *PTGS2* | TAAGTGCGATTGTACCCGGAC | TTTGTAGCCATAGTCAGCATTGT |
| *RARB* | ATGGAGGTGACCTACCCAGT | AACTACAGACACCAGACGGC |
| *RIPK1* | TGGGCGTCATCATAGAGGAAG | CGCCTTTTCCATGTAAGTAGCA |
| *RIPK2* | CGCTGCTCGACAGTGAAAGAA | GCAGGATGCGAAATCTCAATGG |
| *RNASE7* | CCAAGGGCATGACCTCATCAC | ACCGTTTTGTGTGCTTGTTAATG |
| *RPL13A* | AAGGTGGTGGTCGTACGCTGTG | CGGGAAGGGTTGGTGTTCATCC |
| *S100A4* | CACAAGTACTCGGGCAAAGA | TACACATCATGGCGATGCAG |
| *S100A8* | TTTCCATGCCGTCTACAG | ACGCCCATCTTTATCACC |
| *SCGB1A1* | ACATGAGGGAGGCAGGGGCTC | ACTCAAAGCATGGCAGCGGCA |
| *SERPINB1* | TCAGCTTGCCCAGGTTCAAACTG | GGATGCTACCTGAGGAATTATGC |
| *SFTPD* | AAGCAGGGGAACATAGGACCT | ACACCTCGCTCTCCCTTAGG |
| *SLPI* | GAGATGTTGTCCTGACACTTGTG | AGGCTTCCTCCTTGTTGGGT |
| *SNAI1* | TCGGAAGCCTAACTACAGCGA | AGATGAGCATTGGCAGCGAG |
| *SNAI2* | TGTGACAAGGAATATGTGAGCC | TGAGCCCTCAGATTTGACCTG |
| *TJP1* | GACCAATAGCTGATGTTGCCAGAG | TGCAGGCGAATAATGCCAGA |
| *TGFB1* | CTAATGGTGGAAACCCACAACG | TATCGCCAGGAATTGTTGCTG |
| *TGFB2* | CAGCACACTCGATATGGACCA | CCTCGGGCTCAGGATAGTCT |
| *TGFB3* | ACTTGCACCACCTTGGACTTC | GGTCATCACCGTTGGCTCA |
| *TIGIT* | TCTGCATCTATCACACCTACCC | CCACCACGATGACTGCTGT |
| *TIMP1* | ACCATGGCCCCCTTTGA | CAGCCACAGCAACAACAGGAT |
| *TLR2* | TCTCGCAGTTCCAAACATTCCAC | TTTATCGTCTTCCTGCTTCAAGCC |
| *TLR3* | TTGCCTTGTATCTACTTTTGGGG | TCAACACTGTTATGTTTGTGGGT |
| *TLR4* | TTATCCAGCACACGAATACACAG | AGGCATCTGGTAGAGTCATCAA |
| *TLR5* | TCCCTGAACTCACGAGTCTTT | GGTTGTCAAGTCCGTAAAATGC |
| *TNFA* | AGCCCATGTTGTAGCAAACC | ATGAGGTACAGGCCCTCTGAT |
| *TP63* | CCACCTGGACGTATTCCACTG | TCGAATCAAATGACTAGGAGGGG |
| *TSLP* | ATGTTCGCCATGAAAACTAAGGC | GCGACGCCACAATCCTTGTA |
| *TUBA1A* | TCGATATTGAGCGTCCAACCT | CAAAGGCACGTTTGGCATACA |
| *TWIST1* | GTCCGCAGTCTTACGAGGAG | GCTTGAGGGTCTGAATCTTGCT |
| *VEGF* | CGAGGGCCTGGAGTGTGT | TGGTGAGGTTTGATCCGCATA |
| *VIM* | TTGAACGCAAAGT​GGAATC | AGGTCAGGCTTGGAAACA |
| *WNT5B* | ACGCTGGAGATCTCTGAGGA | CGAGGTTGAAGCTGAGTTCC |
