# Supplementary figures and images for: Airway epithelial cells mount an early response to mycobacterial infection
Source: Front Cell Infect Microbiol. 2023 Sep 26;13:1253037. doi: 10.3389/fcimb.2023.1253037 (PMC10562574; doi:10.3389/fcimb.2023.1253037)

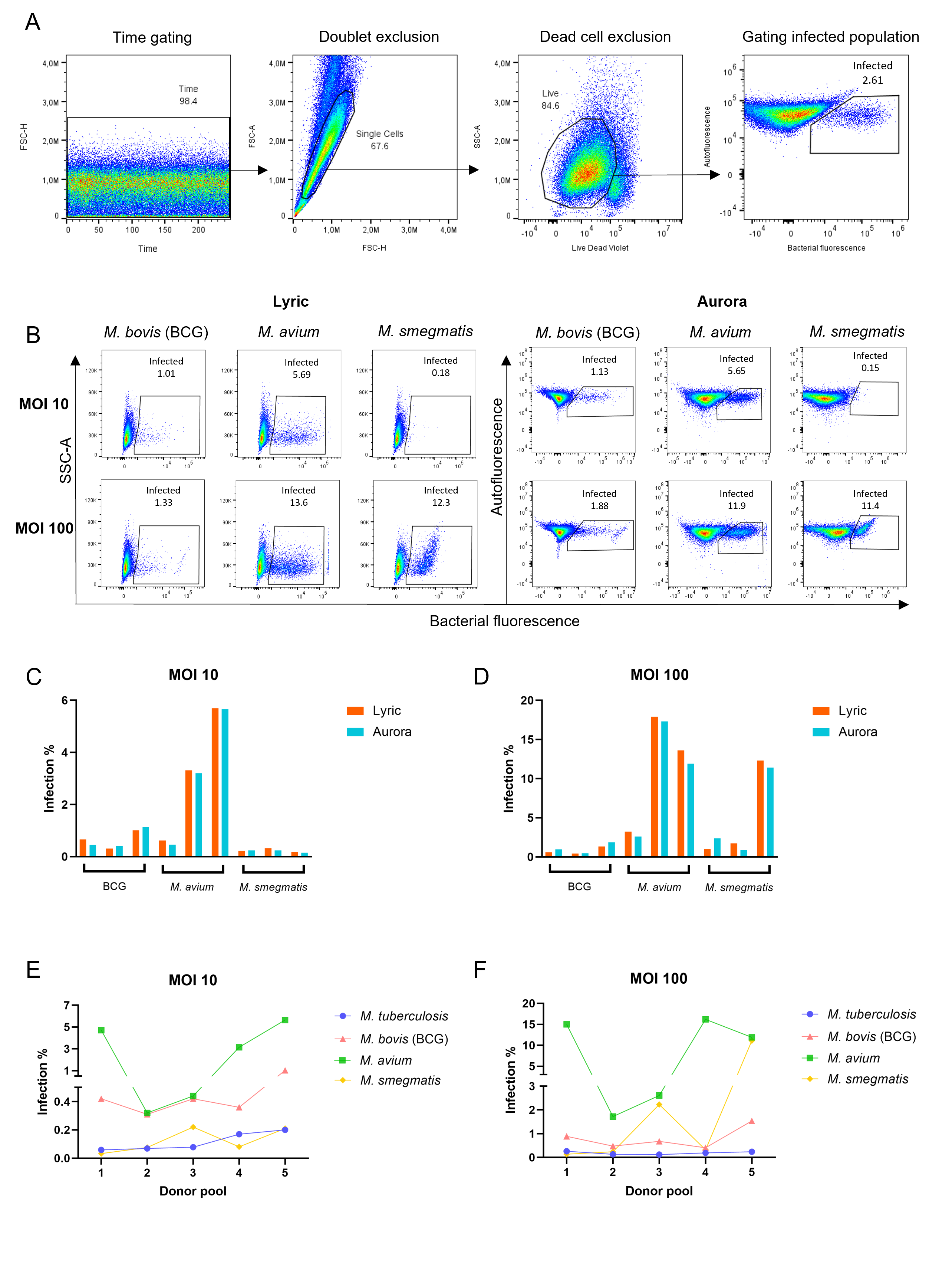

Supplement: Supplementary Figure 1 — Flow cytometry gating strategies and comparison between flow cytometers. (A) Flow cytometry gating strategy to determine the percentage of mycobacteria-infected cells in the ALI-PBEC cultures. (B) Representative result of one flow cytometry experiment measuring the same samples on two different cytometers. (C, D) Comparison of percentage of infected cell in ALI-PBEC cultures infected with MOI 10 and 100 (N=3 independent experiments with different donor pools) between two cytometers. (E, F) Percentage infected cells of ALI-PBEC cultures infected with various mycobacterial species at MOI 10 and 100. Data are depicted per pathogen and per donor pool (n=5). [file Image_1.tif]

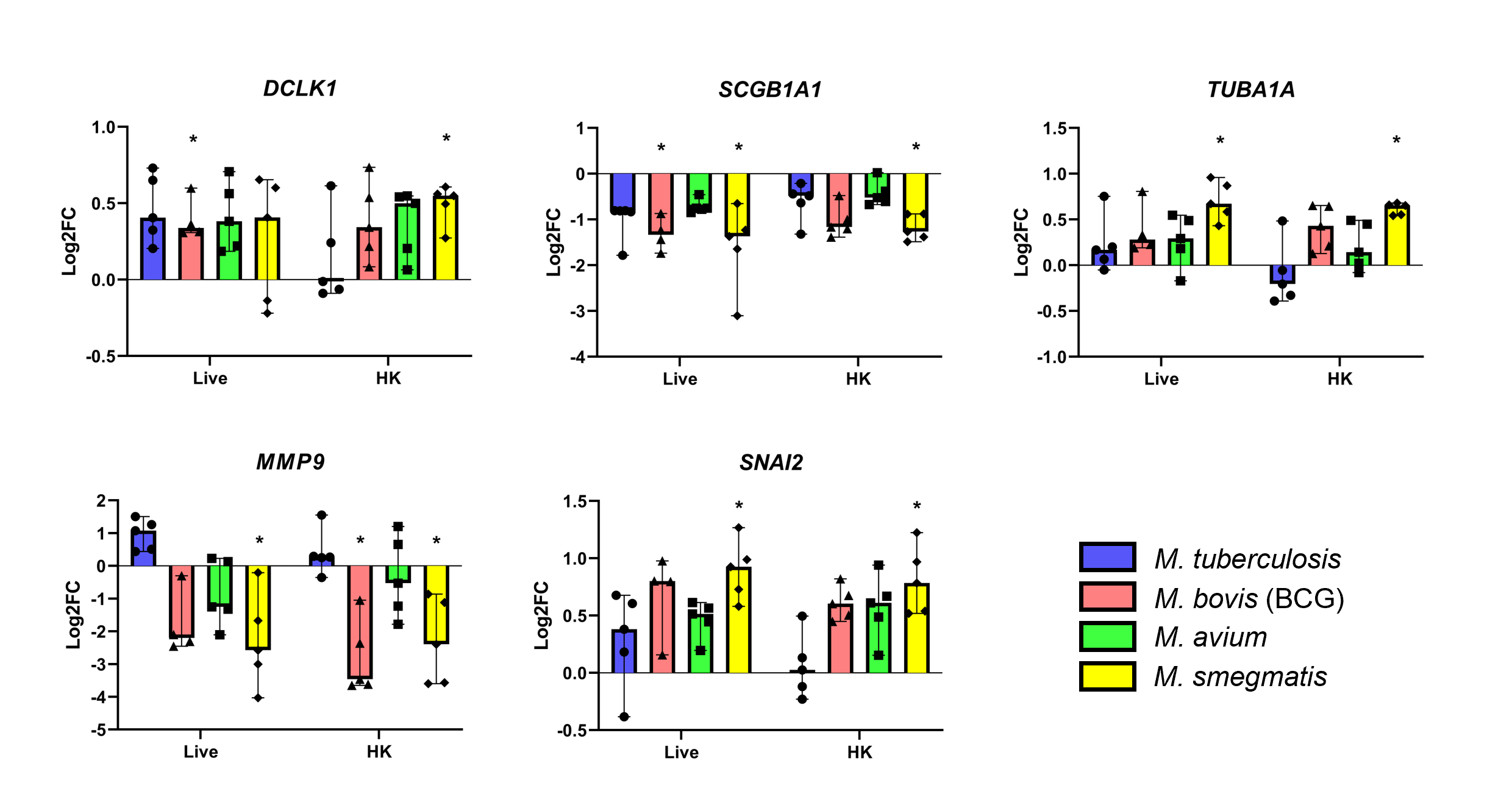

Supplement: Supplementary Figure 2 — Pathogen-specific differential gene expression patterns induced by mycobacterial infection. Bar graphs depicting pathogen-specific induction of differential gene expression in ALI-PBEC cultures infected with four mycobacterial species. Statistical significance determined by Mann-Whitney U test. [file Image_2.tif]

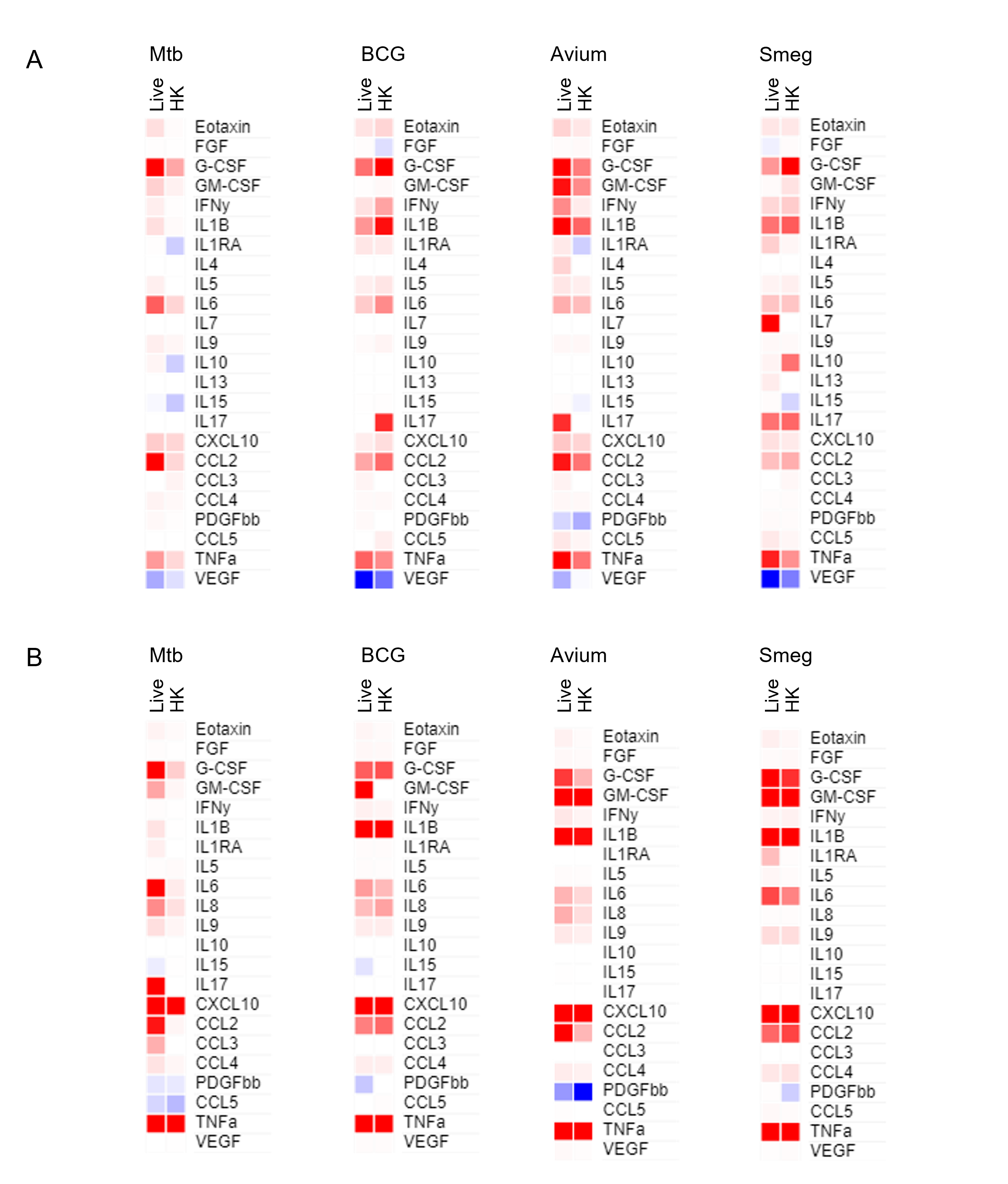

Supplement: Supplementary Figure 3 — Comparison of cytokine secretions induced by live or heat-killed mycobacteria in infected primary bronchial epithelial cells. (A) Heat maps depicting apically secreted cytokines by PBEC after infection with live and heat-killed mycobacteria. Changes in secretion were determined by comparing the median fold change to secretion by uninfected PBEC of 5 donor pools. (B) Heat maps depicting basally secreted cytokines by PBEC after infection with live and heat-killed mycobacteria. Changes in secretion were determined by comparing the median fold change to secretion by uninfected PBEC of 5 donor pools. [file Image_3.tif]

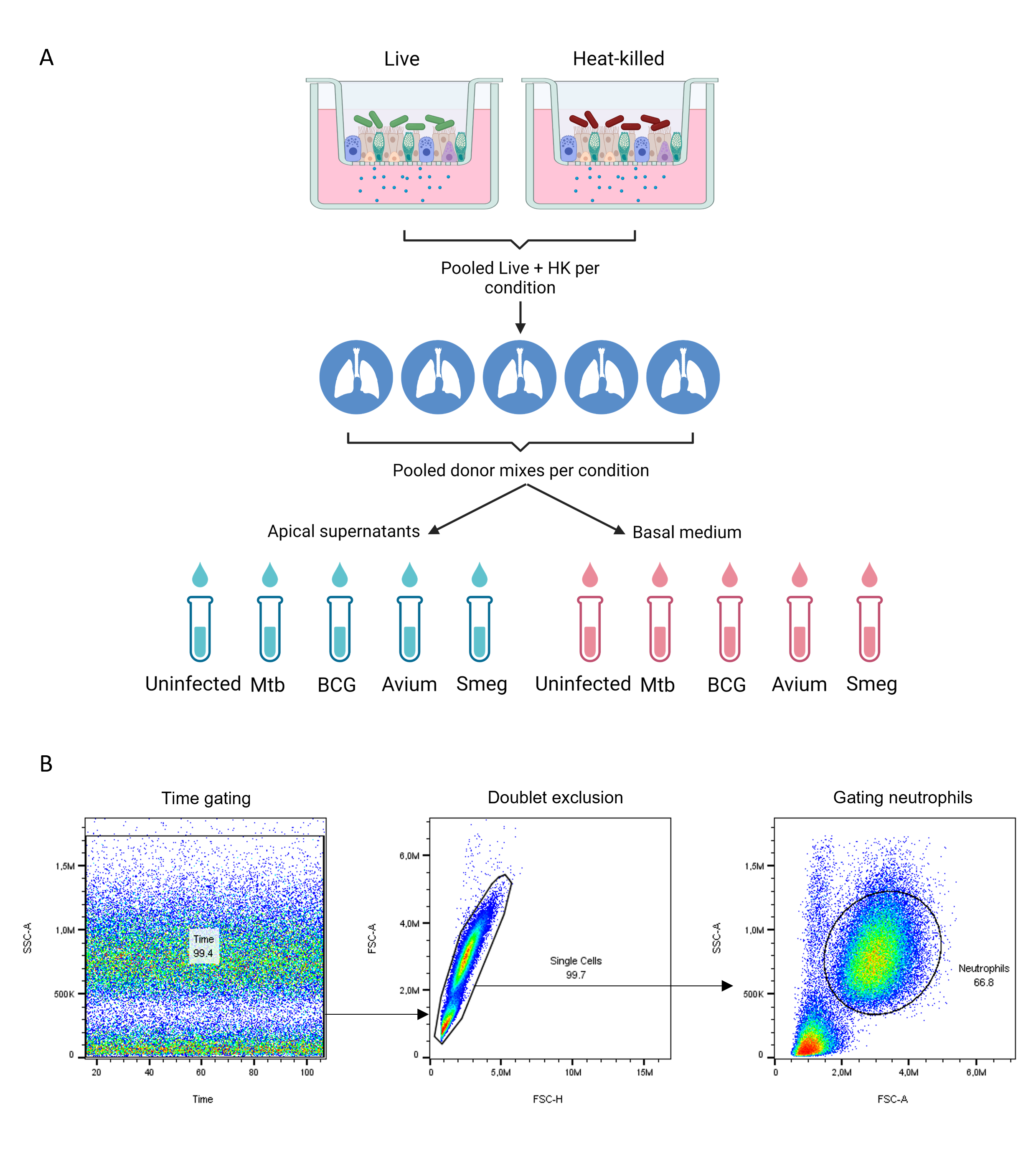

Supplement: Supplementary Figure 4 — Sample pooling strategy and flow cytometry gating strategy for neutrophil migration assay. (A) Schematic showing sample pooling strategy for apical wash and basal medium samples from infected ALI-PBEC. Pooled samples were used in the neutrophil migration assay. Created with BioRender.com. (B) Flow cytometry gating strategy to determine counts of neutrophils that migrated in response to apical wash and basal medium samples from infected ALI-PBEC. [file Image_4.tif]
